# Pain management via Ultrasound-guided Nerve Block in Emergency Department; a Case Series Study

**Published:** 2017-01-09

**Authors:** Amir Nejati, Houman Teymourian, Leili Behrooz, Gholamreza mohseni

**Affiliations:** 1 Emergency Medicine Department, Imam Khomeini Hospital, Tehran University of Medical Sciences, Tehran, Iran.; 2 Department of Anesthesiology, Shohada-e-Tajrish Hospital, Shahid Beheshti University of Medical Sciences, Tehran, Iran.

**Keywords:** Nerve Block, Ultrasonography, Interventional, Pain Management, Emergency Service, Hospital

## Abstract

**Introduction:**

Pain is the most common complaint of patients referring to emergency department (ED). Considering the importance of pain management in ED, this study aimed to investigate the efficacy and feasibility of ultrasound-guided nerve blocks in this setting.

**Methods:**

46 patients who came to the ED with injured extremities were enrolled in the study and received either femoral, axillary or sciatic nerve block depending on their site of injury (1.5 mg Bupivacaine per kg of patient’s weight). Patients were asked about their level of pain before and after receiving the nerve block based on numerical rating scale. The difference between pre and post block pain severity was measured. Both patients and physicians were asked about their satisfaction with the nerve block in 5 tiered Likert scale.

**Results:**

46 patients with the mean age of 37.5 ± 12.5 years (8-82 years) received ultrasound-guided nerve block (84.8% male). 6 Sciatic, 25 axillary, and 15 femoral nerve blocks were performed. Mean pain severity on NRS score at the time of admission was 8.1 ± 1.4, which reduced to 2.04 ± 2.06 after block. 25 (54.3%) patients were highly satisfied (Likert scale 5), 15 (32.6%) were satisfied (Likert scale 4), 3 (6.5%) were neutral and had no opinion (Likert scale 3), 1 (2.1%) was not satisfied (Likert scale 2), and 2 (4.3%) were highly unsatisfied (Likert scale 1). There was no significant difference among the satisfaction scores within the three block locations (p = 0.8). There was no significant difference in physicians’ level of satisfaction between the three block locations either (p = 0.9). 1 (2.1%) case of agitation and tachycardia and 1 (2.1%) case of vomiting were observed after the procedure.

**Conclusion:**

Ultrasound-guided nerve block of extremities is a safe and effective method that can be used for pain management in the ED. It results in high levels of satisfaction among both patients and physicians.

## Introduction

Pain is the most common complaint of patients presented to the emergency department (ED) (1). In addition, many ED procedures, such as fracture reduction, need local anesthesia to gain patient’s cooperation during the procedure. Procedural sedation and analgesia (PSA) require airway and hemodynamic monitoring, which may be time-consuming for physicians and the ED staff. PSA has rare yet serious side effects such as hypotension, allergic reactions, and respiratory compromise. 

In this regard, nerve blocks can be used as effective and safe tools for pain management in ED as it does not require airway monitoring or long-term care. Hematoma, infection, and pneumothorax are among its complications. However, using the ultrasound-guided method reduces these side effects (2, 3).

Physicians’ challenges in finding anatomical landmarks and limitations of special devices such as electric stimulators of nerves are a few of the reasons behind the limited use of this technique in ED.

Ultrasound-guided nerve blocks were first introduced in anesthesiology in 1978 when La Grange et al. used a Doppler device for performing a supraclavicular block of the brachial plexus (4). However, it was later in 1994 that ultrasound was used for showing the exact location for injection of the anesthetic in the area around a nerve (5).

In the previous studies on patients with extremity injury, the results have shown high efficacy of nerve blocks and no serious side effects have been reported (2, 6, 7). In 2005, in a study by Liebmann et al., radial, ulnar and median nerve blocks with ultrasound guide were done by emergency physicians. Their results showed that all of the procedures (100%) were completed without need for extra anesthetic agent. 92% of the patients indicated that they would want to receive nerve blocks again if pain control was needed. They concluded that specialists, fellows and residents can perform ultrasound-guided nerve blocks, successfully (6).

In one controlled trial, nerve block was used for pain control in femoral neck fractures in the ED of Rotherham General Hospital. Patients who received nerve block reported less time to achieve the lowest level of pain (8).

Beaudoin et al. did a study on femoral nerve block in 2010 and concluded that femoral nerve block with ultrasound guide is effective and useful for pain management in the ED (7).

Nerve block is a relatively new concept in the ED. Considering the importance of pain management in ED and the limited work on ultrasound-guided nerve blocks in Iran, this study aimed to investigate the efficacy and feasibility of this technique in the ED. 

## Methods

***Study setting***

This study was conducted in the ED of an academic Hospital by the emergency medicine physician. The study design was approved by the ethics committee of Tehran University of Medical Sciences and all researchers adhered to all principles of Helsinki declaration during the study period. After thorough explanation of the procedure, written informed consent was signed by all enrolled patients.

***Participants***

Patients who had pain in their upper or lower extremities following trauma and injury, with pain severity higher than or equal to 4, based on numeric rating scale (NRS), were included. Finding a neurological defect in physical examination; planning to transfer the patient to the operation room within 12 hours; allergic history to local anesthetics; infection, hematoma or active bleeding at the site of injection; closed fractures (due to the risk of compartment syndrome); unstable vital signs or Glasgow coma scale < 15; using narcotic agents in the past 24 hours; pregnancy; moderate to severe head trauma with prolonged vomiting, severe progressive headache, rhinorrhea, otorrhea and bleeding from ear and nose; serious thoracic, abdominal or vertebral injuries; speech difficulties; intoxication with alcohol and drugs; mental retardation; and history of seizure were among the exclusion criteria.

***Data gathering***

Patients’ characteristics (age, sex), medical history (history of narcotic use within the past 24 hours and allergy), and vital signs (heart rate and blood pressure) were taken and recorded at the time of admission. A complete physical examination of the injured extremity was performed and the patient’s pain was measured before (baseline), and one hour after the procedure based on NRS. Bupivacaine with maximum dose of 1.5 mg/kg was used for nerve block and distilled water was used to reach the desired volume (50 cc for axillary, 40 cc for femoral and 40 cc for sciatic nerve block). Under sterile conditions, using a 20 or 22 gauge lumbar puncture needle, 50 cc syringe and an extension tube, ultrasound-guided nerve block was performed by a trained emergency physician according to the standard guidelines. A linear probe with a frequency of 7.5 MHz was used for ultrasonography of nerve block locations. One hour after the nerve block, patients’ blood pressure and heart rate were checked again. 

Satisfaction of patient and physician from the procedure was recorded based on 5 tiered Likert scale. Satisfaction rate ranged from 5 meaning highly satisfied to 1 meaning highly unsatisfied. Patients were monitored for two hours and possible complications such as nausea/vomiting, seizure, headache, hypotension and any other complications were recorded. 

***Statistical analysis***

All the data were entered into SPSS 21. First, descriptive characteristics and frequencies were calculated. Continuous variables were reported as mean ± standard deviation and categorical ones as frequency and percentage.

## Results

46 patients with the mean age of 37.5 ± 12.5 years (8-82 years) received ultrasound-guided nerve block (84.8% male). 6 Sciatic, 25 axillary, and 15 femoral nerve blocks were performed. Mean pain severity on NRS score at the time of admission was 8.1 ± 1.4, which reduced to 2.04 ± 2.06 after block. 25 (54.3%) patients were highly satisfied (Likert scale 5), 15 (32.6%) were satisfied (Likert scale 4), 3 (6.5%) were neutral and had no opinion (Likert scale 3), 1 (2.1%) was not satisfied (Likert scale 2), and 2 (4.3%) were highly unsatisfied (Likert scale 1). There was no significant difference among the satisfaction scores within the three block locations (p = 0.8). There was no significant difference in physicians’ level of satisfaction between the three block locations either (p = 0.9). 

Mean systolic blood pressure of patients before and after nerve block were 125.3 ± 5.3 and 119.7 ± 4.8 mmHg, respectively (p < 0.001). Also, mean pulse rate of patients before and after nerve block were 94.76 ± 4.4 and 87.23 ± 5.6, respectively (p < 0.001). 1 (2.1%) case of agitation and tachycardia and 1 (2.1%) case of vomiting were observed after the procedure.

## Discussion

This prospective case series study concluded that ultrasound-guided nerve block, decreases pain by more than 75% and reduces patients’ pain from severe (NRS>8) to tolerable (NRS=2) with minimal side effects. Both physicians and patients were more than 80% satisfied with pain reduction using this method regardless of the block location. Although there was a significant reduction in blood pressure and heart rate after receiving the nerve block, it was not clinically important.

There are many similar studies on the efficacy of proximal nerve blocks in pain reduction for patients, especially performing nerve block under ultrasound guide, which leads to more precision and fewer side effects (9-17). The efficacy of the nerve block is enough to even perform serious surgeries on the patients (9, 11, 14).

Wang et al. concluded in their study that ultrasound-guided nerve block is a better pain reduction method than epidural analgesia (12). A few other studies, in which ultrasound-guided nerve block and PSA were compared, stated that performing nerve block takes less time, patient needs shorter monitoring and observation period and there are also fewer side effects compared to sedation (12, 17, 18).

Various side effects have been reported for nerve blocks, which can be divided to local and systemic.

Local side effects are the side effects that manifest due to needle at the site of injection. Some of these side effects such as arterial puncture, hematoma at the site of injection, infection, phlebitis and thrombosis are common among all types of nerve blocks. Some of the other side effects depend on the anatomic site of the nerve block and its technique as well as the experience of the person performing the nerve block. These include side effects such as: pneumothorax/hemothorax (in axillary, infraclavicular, supraclavicular and rarely suprascalene nerve block) and arteriovenous fistula.

Systemic side effects can happen because of direct injection of anesthetic into the artery or vein which are very rare, and about 0 with ultrasound guide. The incidence of such side effects may increase due to frequent attempts for finding the nerve, injury to the artery or vein, and using the blind method (without ultrasound guide) or with nerve stimulator (16). 

In our study, one patient got irritable and agitated, and developed tachycardia, which was controlled with minor intervention (2 mg of Midazolam was administered and the patient calmed down afterwards).

There was one report of nausea and vomiting, which was relieved without any interventions. In one study, nausea due to nerve block was reported to be much lower than nausea after sedation (12).

In this study, 40 (87%) patients were satisfied with the results (very satisfied or satisfied) and three patients (6.4% of all the patients) were not satisfied. The strong existing correlation between the level of pain and patient satisfaction seems logical considering the effectiveness of nerve block in reducing their pain. Cases of the three dissatisfied patients were investigated. One of them had a deep massive laceration on his shin in addition to femoral fracture which was the reason he got nerve block, this patient did not receive sciatic nerve block at the same time and this was disappointing to the patient since the pain from the laceration was not relieved and so he was unhappy with the procedure. Pain of the other two patients was not relieved after the block, which led to their dissatisfaction. In a study, Luber et al. reported 92% overall satisfaction among patients undergoing lumbar plexus block (19).

In this study, more than 80% of the physicians were satisfied with the results. This can be due to the feasibility of conducting an ultrasound-guided nerve block, patient’s cooperation and the final effects of the procedure. Although physicians’ satisfaction from the three types of nerve blocks were not significantly different, higher levels of satisfaction from axillary nerve block compared to sciatic, could be due to its easier execution or faster identification of the nerve, as for Femoral nerve block it could be because of the proximity of this nerve to the artery which leads to an easier nerve mapping and better access. 

## Limitations:

We did not measure the time spent to perform the nerve block under the guidance of ultrasound. Patients were monitored for two hours after receiving nerve block and their pain was only measured once after the procedure, it would have been better to evaluate their pain more than only once and during a longer period. Considering that we included three types of nerve blocks, the number of our sciatic cases was low and may not have been enough for drawing conclusions. 

In addition, all of the nerve blocks were conducted by one person which decreases the generalizability. 

## Conclusion:

Ultrasound-guided nerve block of extremities is a safe and effective method that can be used for pain management in the ED. It results in high levels of satisfaction among both patients and physicians.
